# Editorial: Current Perspectives on Social Comparisons and Their Effects

**DOI:** 10.3389/fpsyg.2021.739783

**Published:** 2021-10-11

**Authors:** Sviatlana Kamarova, Athanasios Papaioannou, Nikos Chatzisarantis

**Affiliations:** ^1^Future of Work Institute, Curtin University, Perth, WA, Australia; ^2^Department of Physical Education and Sport Science, University of Thessaly, Trikala, Greece; ^3^School of Psychology, Curtin University, Perth, WA, Australia

**Keywords:** social comparisons, selection social comparisons, reaction social comparisons, well-being, performance, self-evaluations

Every day in different contexts and with different purposes we engage in social comparison processes, whether consciously or at subliminal level (e.g., Kahneman and Miller, 1986; Mussweiler and Rüter, 2003). Indeed, social comparisons represent a powerful tool people attend to infer their self-worth or to judge on their abilities by “stacking [oneself] up against the others” (Festinger, 1954). The information retrieved this way is treated as more accurate and objective and strategically useful, especially under tight timelines or in situations of uncertainty (e.g., Corcoran et al., 2011; Lockwood et al., 2012; van Dick et al., 2018). Recently, Gerber et al. (2018) presented a meta-analysis of social comparison research, where they identified mechanisms that enhance the social comparison effects. This work showed that besides manipulation of self through priming, novel information assessment indeed showed a consistent increase in social comparison effects as well as proximity of the standards (perceived relevance, similarity, or identification with the standard). The latter was associated with immediacy or salience of the standard perception of which outweigh general comparison (Buckingham and Alicke, 2002; Zell and Alicke, 2013). Finally, the meta-analytical analysis demonstrated that people generally choose upward comparison (better-off) standards, even when such comparison poses a threat to their self-esteem, bridging their interests, and that these comparisons tend to undermine well-being and ability self-evaluations. According to Gerber et al. (2018), contrast is a default reaction to social comparisons, whereas assimilation appears when conditions that suggest these processes are provided through priming, identification with the standard, or situations of uncertainty. Overall, this evidence only partly confirms the Self-Evaluation Model (SEM; Mussweiler, 2003), which suggested assimilation as a default mechanism and a threat to self-esteem to guide the use of social comparison information not allowing to inflict a traumatic conclusion.

To further the meta-analysis and existing knowledge on social comparisons, the 12 articles comprising this collection, reflect most recent perspectives and trends concerning social comparisons in Psychology and related disciplines, covering a wide range of aspects. First, conceptual and methodological issues were the focus of several papers. In Arigo's et al. scoping review on methods used to assess social comparison processes within persons in daily life argued that an ecological momentary assessment or daily diaries utilised in social and clinical research represent a more powerful and valid method to measurement rather than a traditional aggregated retrospective self-report. Furthermore, Whillans et al. proposed their conceptual framework on the long-term benefits of worse-than-average beliefs in domains including motivation, task performance, and subjective well-being, which generates novel insights in skill learning and provides recommendations for future research. In their conceptual paper, Caricati and Owuamalam argued that social comparison processes can act as a tool allowing justification of the existing societal systems where intermediately positioned disadvantaged through downward assimilation to the worse off.

Experimental and applied research on health and well-being examined specific issues and the mechanism by which social effects are derived. Specifically, Corcoran et al. found that social comparisons can be beneficial for cancer patients if they engage in the right process by engaging in downward comparison processes by contrasting from poorly adjusted patients and assimilating to well-adjusted ones. Interestingly, Arnold et al. also found that downward social or temporal comparisons (i.e., evaluated their contact with others as better-off) related to lower loneliness levels compared to upward comparisons, even when controlling for baseline levels. Furthermore, Wayment et al. provided evidence in support of social comparison processes and their functionality: lateral (similarity) and upward social comparisons were instrumental for meeting accuracy and self-improvement motives during weight loss, while for the self-enhancement motive were lateral and downward social comparisons. In application to the population of women with fibromyalgia, Cantero et al. found that patients with higher level of pain perception, anxiety and depression attend to more disadvantage types of comparison such as upward contrast and downward identification as opposed to those with lower levels of pain perception, anxiety and depression use upward identification and downward contrast. In a 2.5-year longitudinal study, Brycz et al. found that individuals with a larger insight for their biases (stronger metacognitive self) sought more social comparisons information, of both directions, for self-improvement purposes. Next, the moderating effects of athletic mental energy on the athletes' life stress–burnout relationship was examined by Chiou et al., as an ability to ignore social comparisons in competitive environment buffering debilitating effects for well-being.

Finally, several studies examined social comparison processes and effects in relation to performance and decision-making. For example, Akay et al. found that empathy, defined via its affective and cognitive aspects, cause positional concerns (i.e., choices), positively relating to self-gain choices and negatively relating to choices reflecting losing (other gain). Taking an organisational psychology perspective, Sijbom and Parker found that leaders who attend to self-referenced standards (mastery-approach goals) during self-evaluations were more receptive to their subordinates, while leaders who base their self-evaluations on social comparisons (performance-approach goals) were less receptive to their subordinates in threatening situations of low power. Finally, Dolean and Cãlugãr demonstrated that SES-driven social comparison processes can explain most of the inter-ethnic differences in general non-verbal intellectual abilities (IQ measured with Raven Progressive Matrices) in a Roma ethnic minority in Rumania, indicating that Roma's students poor performance on such tests is not a true reflection of the population mean. In line with social comparison theory (Festinger, 1954) and SEM (Mussweiler, 2003), the identity processes linked to in-out-Roma-group can produce lower performance on IQ tests by means of unfavourable effects of downward assimilation with lower performing children (Roma group) and contrast from higher performing ones (non-Roma group), increasing the ethnic separation.

The current collection of articles presents different takes on social comparisons, their nature and effects they produce, and we hope that this special issue will be of interest to researchers from a variety of fields, practitioners and policy makers.

## Author Contributions

SK and NC contributed to the conceptualisation of the special issue. SK, NC, and AP reviewed and edited articles included in the issue, and contributed to the editorial at its different stages.

## Conflict of Interest

The authors declare that the research was conducted in the absence of any commercial or financial relationships that could be construed as a potential conflict of interest.

## Publisher's Note

All claims expressed in this article are solely those of the authors and do not necessarily represent those of their affiliated organizations, or those of the publisher, the editors and the reviewers. Any product that may be evaluated in this article, or claim that may be made by its manufacturer, is not guaranteed or endorsed by the publisher.
